# Transition of Emission Colours as a Consequence of Heat-Treatment of Carbon Coated Ce^3+^-Doped YAG Phosphors

**DOI:** 10.3390/ma10101180

**Published:** 2017-10-16

**Authors:** Liang-Jun Yin, Benjamin Dierre, Takashi Sekiguchi, J. Ruud van Ommen, Hubertus T. (Bert) Hintzen, Yujin Cho

**Affiliations:** 1 School of Energy Science and Engineering, University of Electronic Science and Technology of China, 2006 Xiyuan Road, Chengdu 610051, China; 2 Department of Chemical Engineering, Faculty of Applied Sciences, Delft University of Technology, Van der Maasweg 9, 2629 HZ Delft, The Netherlands; J.R.vanOmmen@tudelft.nl; 3 Luminescent Materials Research Group, Faculty of Applied Sciences, Delft University of Technology, Mekelweg 15, 2629 JB Delft, The Netherlands; h.t.hintzen@tudelft.nl; 4 Semiconductor Device Materials Group, International Center for Materials Nanoarchitectonics (MANA), National Institute for Materials Science (NIMS), 1-1 Namiki, Tsukuba, Ibaraki 305-0044, Japan; sekiguchi.takashi@nims.go.jp

**Keywords:** YAG:Ce^3+^, phosphors, luminescence, carbon coating, electron microscopy, cathodoluminescence, heat treatment

## Abstract

To modify the luminescence properties of Ce^3+^-doped Y_3_Al_5_O_12_ (YAG) phosphors, they have been coated with a carbon layer by chemical vapor deposition and subsequently heat-treated at high temperature under N_2_ atmosphere. Luminescence of the carbon coated YAG:Ce^3+^ phosphors has been investigated as a function of heat-treatment at 1500 and 1650 °C. The 540 nm emission intensity of C@YAG:Ce^3+^ is the highest when heated at 1650 °C, while a blue emission at 400–420 nm is observed when heated at 1500 °C but not at 1650 °C. It is verified by X-ray diffraction (XRD) that the intriguing luminescence changes are induced by the formation of new phases in C@YAG:Ce^3+^-1500 °C, which disappear in C@YAG:Ce^3+^-1650 °C. In order to understand the mechanisms responsible for the enhancement of YAG:Ce^3+^ emission and the presence of the blue emission observed for C@YAG:Ce^3+^-1500 °C, the samples have been investigated by a combination of several electron microscopy techniques, such as HRTEM, SEM-CL, and SEM-EDS. This local and cross-sectional analysis clearly reveals a gradual transformation of phase and morphology in heated C@YAG:Ce^3+^ phosphors, which is related to a reaction between C and YAG:Ce^3+^ in N_2_ atmosphere. Through reaction between the carbon layer and YAG host materials, the emission colour of the phosphors can be modified from yellow, white, and then back to yellow under UV excitation as a function of heat-treatment in N_2_ atmosphere.

## 1. Introduction

Yttrium aluminum garnet Y_3_Al_5_O_12_ (YAG) is widely used as an optical host material due to its remarkable chemical stability, good mechanical properties, and flexible structural compatibility [1,2,3]. Many rare-earth or transition ions (Ce^3+^, Tb^3+^, Yb^3+^, Eu^3+^, Tm^3+^, Ho^3+^, Cr^3+^, etc.) doped YAG materials have been reported, and applied in the illumination and display field [4,5,6,7,8]. Especially, white light can be generated through a simple combination of blue-emitting devices and yellow emitting phosphor YAG:Ce^3+^, i.e., phosphor-converted LEDs (PC-LEDs), which have been successfully commercialized [9]. Therefore, adjusting luminescence properties of YAG:Ce^3+^ attract much attention via codoping with another lanthanide ions (La^3+^, Pr^3+^, Tb^3+^, Sm^3+^ codoping) [10,11,12,13,14] or modification of the host-lattice by substitution (Lu/Y, Ga/Al, Si/Al, N/O, Si-N/Al-O) [15,16,17,18,19,20].

In practical applications, the phosphors in PC-LEDs may undergo thermal degradation after operating for a long time at a temperature higher than 150 °C [21]. In our previous work, it has been demonstrated that carbon coating deposited on the surface of BaMgAl_10_O_17_:Eu^2+^ phosphors is an effective way to improve the intensity and thermal stability of oxide phosphors [22]. The carbon coated phosphors show a higher emission intensity and a better oxidation resistance at high temperature than uncoated phosphors. These results also motivate us to perform carbon deposition on the surface of YAG:Ce^3+^ and investigate the effect of post heat-treatment on the structure and luminescence properties of YAG:Ce^3+^.

Referring to the carbothermal reduction and nitridation in the synthesis of nitrides from oxides and C in N_2_ atmosphere [23,24,25], we have applied post heat-treatment of carbon coated YAG:Ce^3+^ in N_2_ atmosphere in order to check whether it possibly triggers a reaction between C and YAG. Hence, a change in phase composition and microstructure may be introduced into YAG:Ce^3+^, which influences the luminescence properties of YAG:Ce^3+^. In addition, it is believed that the reduction ability is more significant in carbon-containing atmosphere than only in N_2_ atmosphere. When performing post heat-treatment on carbon coated YAG in N_2_ atmosphere, carbon will create a strong reducing atmosphere, converting the Ce^4+^ residual in YAG into Ce^3+^ ions based on the following formula [26], which is beneficial for the enhanced luminescence intensity of YAG:Ce^3+^ phosphor.

$${2\mathrm{CeO}}_{2}+C\to {\mathrm{Ce}}_{2}O_{3}+\mathrm{CO}$$

In this paper, we investigate the deposition of a thin carbon layer on the surface of YAG:Ce^3+^ phosphors, and then performed heat-treatment of carbon coated YAG:Ce^3+^ phosphors in N_2_ atmosphere. It is demonstrated that a reaction between C and YAG indeed happens, which leads to changes in emission colour under ultraviolet (UV) irradiation as a function of different heat-treatment temperature. 

## 2. Experimental Details

YAG:Ce^3+^ phosphors with a composition of Y_2.94_Ce_0.06_Al_5_O_12_ were prepared by solid-state reaction in Boron Nitride (BN) crucible from starting mixtures of Y_2_O_3_, Al_2_O_3_, and CeO_2_ (99.99 wt %, Sinopharm Chemical Reagent Co. Ltd. Shanghai, China) in N_2_ atmosphere at 1500 °C for 4 h. Then, carbon coating on the surface of YAG: Ce^3+^ was performed by chemical vapor deposition (CVD) at 700 °C under gas mixtures of N_2_ (gas flow rate: 100 mL/min) and C_2_H_2_ (gas flow rate: 50 mL/min). The procedures are as follows: (i) YAG:Ce^3+^ phosphor was put in the quartz boat; (ii) The quartz boat was put inside a conventional horizontal tube furnace. The furnace was heated at 700 °C for 30 min under N_2_ atmosphere (gas flow rate: 100 mL/min); (iii) C_2_H_2_ gas (gas flow rate: 50 mL/min) was introduced into the tube for 5 min; (iv) After natural cooling of the furnace under N_2_ atmosphere, carbon-coated YAG:Ce^3+^(C@YAG:Ce^3+^) was taken out; and, (v) C@YAG:Ce^3+^ was heat treated in BN crucible at 1500 and 1650^o^C for 2 h in N_2_ atmosphere (C@YAG:Ce^3+^-1500 °C, C@YAG:Ce^3+^-1650 °C). As a comparison, a similar procedure of heat-treatment was carried out for the uncoated YAG:Ce^3+^. The obtained powders were milled in Si_3_N_4_ mortar by hand for further measurements. 

Photoluminescence spectra (PL) were measured by a fluorescence spectrophotometer (Model F-4600, Hitachi, Tokyo, Japan) with a 200 W Xe lamp as an excitation source. The emission spectrum was corrected for the spectral response of the monochromator and photomultiplier tube (R928P, Hamamatsu Photonics K.K., Hamamatsu, Japan) by a light diffuser and tungsten lamp (Noma Electric Corp., New York, NY, USA; 10 V, 4 A). The photoluminescence excitation (PLE) spectrum was also corrected for the spectral distribution of the Xe lamp intensity by measuring Rhodamine-B as a reference. 

The phase formation was analyzed by X-ray diffraction (XRD; Model PW 1700, Philips, Eindhoven, The Netherlands) using Cu Kα radiation at a scanning rate of 2°/min. The microstructure was observed by using High-Resolution Transmission Electron Microscopy (HRTEM; 2100F, JEOL, Tokyo, Japan). Energy-Dispersed Spectroscopy (EDS) was done at room temperature in a high-resolution-field emission Scanning Electron Microscope (SEM; SU8000, Hitachi, Tokyo, Japan). SEM and cathodoluminescence (CL) measurements were performed in a field emission SEM (S4300, Hitachi) equipped with a CL system (MP32S/M, Horiba, Kyoto, Japan) at 5 kV and room temperature. To perform cross-sectional analysis, mirror surface of phosphor particles were produced by using Ar ion cross section polisher (SM-09010, JEOL Ltd., Tokyo, Japan) after embedded in an epoxy resin (G2, Gatan Inc., Pleasanton, CA, USA) [27].

## 3. Results

### 3.1. IIIa Phase Formation, Microstructure and Morphology

**Figure 1:**
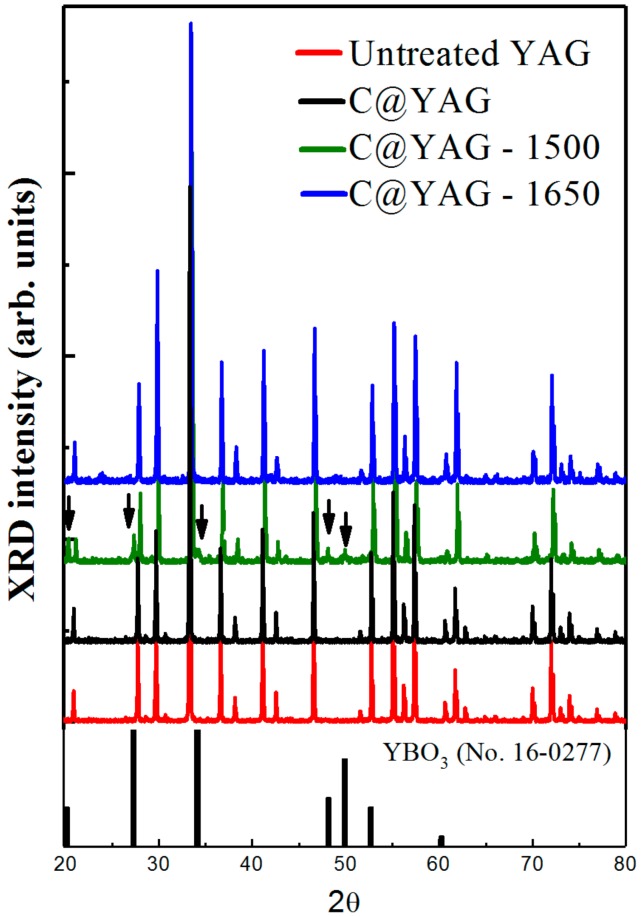
X-ray diffraction (XRD) patterns for untreated YAG:Ce^3+^, C@YAG:Ce^3+^,C@YAG:Ce^3+^-1500 °C and C@YAG:Ce^3+^-1650 °C. The arrows indicate the additional peaks ascribed to secondary phases.

Figure 1 shows the XRD patterns for untreated YAG:Ce^3+^, C@YAG:Ce^3+^, C@YAG:Ce^3+^-1500 °C and C@YAG:Ce^3+^-1650 °C, respectively. All of the peaks are sharp with high intensity, suggesting good crystallinity. Most of the peaks in all of the samples are in good agreement with the standard diffraction peaks of YAG (JCPDS Card No. 33-0040), indicating the main phase is YAG with the garnet phase. No XRD peaks for crystalline carbon are detected in C@YAG:Ce^3+^ samples, suggesting that the carbon film deposited on the surface of YAG:Ce^3+^ is amorphous or very thin. Some additional peaks due to secondary phases are detected for C@YAG:Ce^3+^-1500 °C. Their nature will be discussed in the following. Interestingly, these secondary peaks are not observed for C@YAG:Ce^3+^-1650 °C.

**Figure 2:**
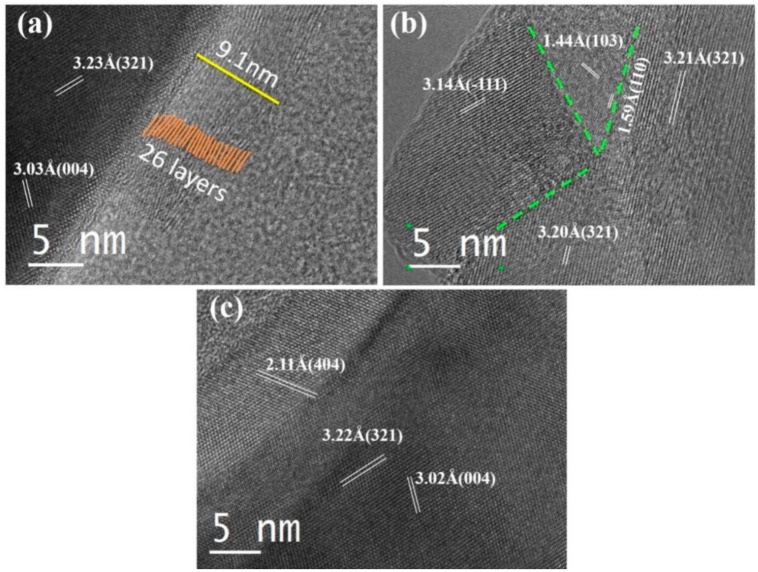
High-Resolution Transmission Electron Microscopy (HRTEM) images for C@YAG:Ce^3+^ (**a**); C@YAG:Ce^3+^-1500 °C (**b**) and C@YAG:Ce^3+^-1650 °C (**c**).

Microstructures of the C@YAG:Ce^3+^, C@YAG:Ce^3+^-1500 °C, and C@YAG:Ce^3+^-1650 °C samples are examined using HRTEM. Through CVD, carbon material has been successfully coated on the surface of YAG:Ce^3+^ phosphor particles (Figure 2). The coating layer is estimated to have a thickness of 9.1 nm, corresponding to 26 atomic layers. The distance between every two layers is approximately 0.35 nm, quite similar to the interlayer spacing in multilayer grapheme [28]. Typical HRTEM images of C@YAG:Ce^3+^ (Figure 2a) show a very uniform contrast inside the YAG host, indicating that the synthesized YAG sample has no extended defects. The identified planes have d-spacings 3.23 and 3.03 Å, indexed as (321) and (004) plane of YAG, respectively, and their planar angle is 74.3°. It should be noted that no C coating layers are observed for both C@YAG:Ce^3+^-1500 °C and C@YAG:Ce^3+^-1650 °C samples. In the C@YAG:Ce^3+^-1500 °C sample, the image reveals obvious multi-domain nanostructures (Figure 2b). Besides the major phase ascribed to YAG, there are other secondary phases present in the C@YAG:Ce^3+^-1500 °C sample. As shown in the XRD patterns of Figure 1, YBO_3_ phase is detected in C@YAG:Ce^3+^-1500 °C as a result of reaction between Y_2_O_3_ and the B_2_O_3_ impurity from BN crucible. d-spacing (3.14 Å), measured from the HRTEM images in the left domain, approximately matches with the value 3.07 Å of YBO_3_ (101) (JCPDS No. 16-0277). d-spacings (1.44 Å and 1.59 Å) and their planar angle (63.2°) calculated in the adjacent domain correspond to the (103) and (110) plane of hexagonal AlN (JCPDS No. 25-1133). Therefore, C@YAG:Ce^3+^-1500 °C sample is not a single-phase phosphor, but consists of YAG, yttrium oxide (i.e., YBO_3_, as also detected in XRD result), and AlN (not detected in XRD result, probably because Al and N are light elements), indicating reaction between YAG host and C in the heat-treatment process at high temperature 1500 °C, proposed as follows.

$$\begin{array}{l} Y_{3}{\mathrm{Al}}_{5}O_{12}+(7.5-3x)C+\left(2.5-x\right)N_{2}\to 1.5Y_{2}O_{3}+{\mathrm{xAl}}_{2}O_{3}+\left(5-2x\right)\mathrm{AlN}+(7.5-3x)\mathrm{CO}\\ Y_{2}O_{3}+B_{2}O_{3}\to 2{\mathrm{YBO}}_{3} \end{array}$$

On account of carbothermal reaction of Al_2_O_3_ to form AlN, the Gibbs equation can be expressed by ΔG = 689.9 × 10^3^ − 0.353 × 10^3^T + RTln ($P_{\mathrm{CO}}^{3}$/$P_{N_{2}}$) [23,29]. In the standard state, the temperature should reach 1680 °C to trigger the reaction. It may be noted that the partial pressure of CO is lower than standard state (P(CO) << 1 atm), making the actual reaction temperature lower than 1680 °C. Therefore, the content of nitrided AlN is low and part of Al_2_O_3_ remains in C@YAG:Ce^3+^-1500 °C. This explains why AlN and Al_2_O_3_ is absent in the corresponding XRD patterns (Figure 1), possibly due to low amount of AlN and Al_2_O_3_ in C@YAG:Ce^3+^-1500 °C. However, only pure YAG phase is observed for C@YAG:Ce^3+^-1650 °C (Figure 2c), indicating the re-dissolution of the yttrium oxide, Al_2_O_3_ and AlN into YAG lattice. It should be noted that the contrast is not uniform, which should be caused by the composition variation of YAG after the segregation-dissolution cycle.

### 3.2. IIIb Average Photoluminescence and Local Cathodoluminescence Properties Related to Chemical Composition and Structure

**Figure 3:**
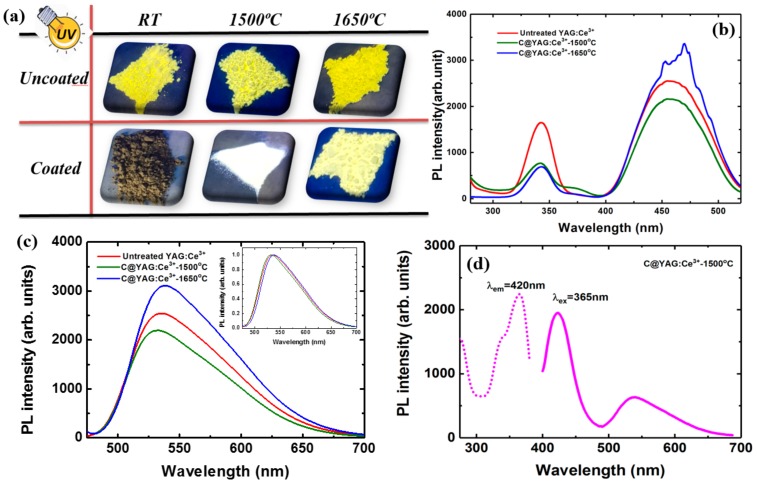
(**a**) Pictures of uncoated and C-coated YAG:Ce^3+^samples under UV-365nm light after heat-treatment at different temperature; (**b**) photoluminescence excitation (PLE) spectra recorded at 540 nm and (**c**) PL spectra under 460 nm excitation for untreated YAG:Ce^3+^, C@YAG:Ce^3+^-1500 °C and C@YAG:Ce^3+^-1650 °C under 460 nm excitation, with inset PL spectra normalized by their respective maxima and (**d**) PLE and PL spectra recorded at different wavelength for C@YAG:Ce^3+^-1500 °C.

It has shown that the phase and composition of YAG:Ce^3+^ can be adjusted via carbon coating and heat-treatment, through which the luminescence properties of YAG:Ce^3+^ can be tuned. To get a direct impression about the effects of heat-treatment on the coated samples, photographs of uncoated and coated samples under 365 nm light are shown in Figure 3a. However, no colour emission changes are observed for the uncoated samples. This was confirmed by PL spectra (Figure S1 in the supporting information). The emission colour is clearly changing for the different coated samples: no emission is observed for C@YAG:Ce^3+^ before heat-treatment; and, C@YAG:Ce^3+^-1500 °C sample exhibits a whitish emission, while C@YAG:Ce^3+^-1650 °C exhibits the yellow emission colour typical for YAG: Ce^3+^. These results suggest that phase and composition tuning, observed by XRD and HRTEM, induces a big transition in luminescence properties. PL measurements on untreated YAG:Ce^3+^, C@YAG:Ce^3+^-1500 °C, and C@YAG:Ce^3+^-1650 °C are shown in Figure 3b,c. All PLE spectrum recorded at 540 nm shows 2 broad bands at 340 and 460 nm, which correspond to the transitions of ^2^F_5/2_ to two lowest lying 5d states, respectively. Under 460 nm excitation, the PL spectra consist of a broad emission with their peaks around 530–540 nm related to the 5d-4f transition of Ce^3+^ in YAG, with the inset showing the normalized PL spectra by their respective maxima. The emission intensity is 2500, 2200, and 3100 for untreated YAG:Ce^3+^, C@YAG:Ce^3+^-1500 °C, and C@YAG:Ce^3+^-1650 °C, respectively. As it can be clearly seen in the inset of Figure 3c, the exact band position is somewhat different for each sample. The emission peak is at 534 nm for untreated YAG:Ce^3+^, 532 nm for C@YAG:Ce^3+^-1500 °C, and 538 nm for C@YAG:Ce^3+^-1650 °C, which may suggest a difference in terms of local environment of Ce^3+^ and/or a change in the valency of Ce ions. According to HRTEM analyses, AlN can be partly produced through carbothermal reaction in N_2_ atmosphere in C@YAG:Ce^3+^-1500 °C. Here, a similar carbothermal reaction could happen for C@YAG:Ce^3+^-1650 °C. With increasing the temperature from 1500 to 1650 °C, formed N will be introduced into YAG lattice and replace the O atom. The replacement of O by N results in a larger crystal field splitting of the 5d energy level of Ce^3+^, and thus a shift of the 5d → 4f emission peak of C@YAG:Ce^3+^-1650 °C to lower energy compared to that of untreated YAG:Ce^3+^ [17,30]. As the XRD pattern of C@YAG:Ce^3+^-1500 °C shows the existence of secondary phases, the excitation wavelength has been changed in order to investigate any possible secondary emission bands. Figure 3d shows the PLE and PL spectra for C@YAG:Ce^3+^-1500 °C. Interestingly, when excited at 365 nm, the PL spectrum consists of a new blue emission band at 420 nm and a weak emission at 532 nm of Ce^3+^ in YAG, which results in a whitish colour. The PLE spectrum taken at 420 nm mainly consists of a band at 365 nm with a shoulder at 340 nm. It has to be noted that the blue emission is only observed for C@YAG:Ce^3+^-1500 °C, and not for C@YAG:Ce^3+^-1650 °C. Similar to untreated YAG:Ce^3+^, C@YAG:Ce^3+^-1650 °C shows a single strong yellow emission. It suggests that the phase composition of C@YAG:Ce^3+^-1500 °C is very different from that of C@YAG:Ce^3+^-1650 °C, which may be ascribed to the carbothermal reaction between YAG and carbon. 

**Figure 4:**
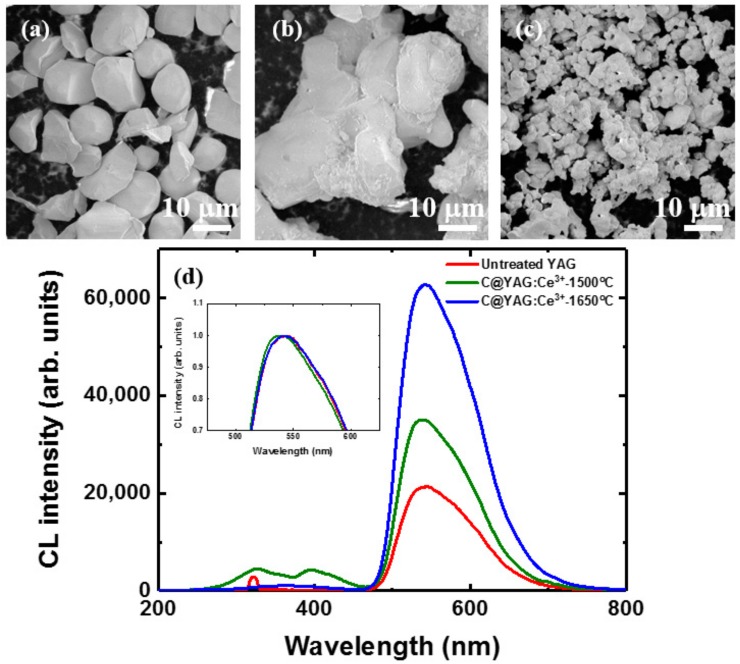
SEM images for untreated YAG:Ce^3+^ (**a**); C@YAG:Ce^3+^-1500 °C (**b**); C@YAG:Ce^3+^-1650 °C (**c**) and (**d**) CL spectra for untreated YAG:Ce^3+^, C@YAG:Ce^3+^-1500 °C, and C@YAG:Ce^3+^-1650 °C, with inset of CL spectra normalized by their respective maxima.

In order to understand in more detail the mechanisms responsible for the presence of the blue emission observed for C@YAG:Ce^3+^-1500 °C and the enhancement of YAG:Ce^3+^ emission for C@YAG:Ce^3+^-1650 °C, we have performed local analysis of different YAG:Ce^3+^ samples by using SEM, CL, and EDS. Figure 4 shows the SEM images for untreated YAG:Ce^3+^ (a), C@YAG:Ce^3+^-1500 °C (b), and C@YAG:Ce^3+^-1650 °C (c). The untreated YAG:Ce^3+^ powders consist of large particles with a diameter of ~10 μm and an apparent smooth surface. The C@YAG:Ce^3+^-1500 °C powders consist of aggregated particles with an apparent size of few tens μm, which may be related to the fusion of several YAG:Ce^3+^ grains. At certain locations, the surface of these particles shows some irregularities, while in others, the surface seems smooth. The C@YAG:Ce^3+^-1650 °C powders have particles with an apparent size of only a few μm, which is much smaller compared to untreated YAG:Ce^3+^ and C@YAG:Ce^3+^-1500 °C. Figure 4d shows the CL spectra of various samples, with inset CL spectra normalized by their respective maxima. The intensity of the 540 nm band is 21,000, 35,000, and 63,000 for untreated YAG:Ce^3+^, C@YAG:Ce^3+^-1500 °C, and C@YAG:Ce^3+^-1650 °C, respectively. However, as it may be seen in the inset, the emission of C@YAG:Ce^3+^-1500 °C and C@YAG:Ce^3+^-1650 °C sample are slightly blue-shifted and red-shifted, respectively, as compared to untreated YAG:Ce^3+^ as similarly observed by PL, suggesting a difference in the local environment of Ce^3+^ or concentration of Ce^3+^. On the other hand, the clearest difference between the samples is observed among the secondary bands in the 250–450 nm range. For untreated YAG:Ce^3+^, a relatively sharp emission is observed at 320 nm, which may be related to Gd^3+^ (315 nm) [31]. The secondary bands are at 330 and 395 nm for C@YAG:Ce^3+^-1500 °C, while at 350 nm for C@YAG:Ce^3+^-1650 °C.

**Figure 5:**
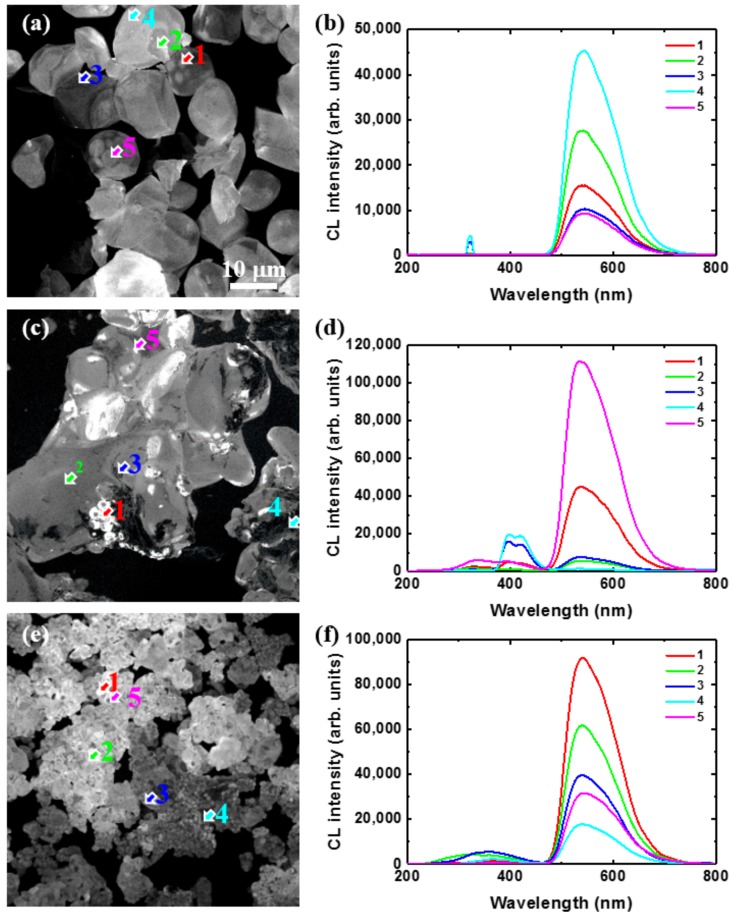
Cathodoluminescence (CL) images taken at 540 nm corresponding to the previous SEM images (**a**,**c**,**e**) and local CL spectra taken on locations indicated by arrows in the images (**b**,**d**,**f**) for untreated YAG:Ce^3+^ (**a**,**b**), C@YAG:Ce^3+^-1500 °C (**c**,**d**) and C@YAG:Ce^3+^-1650 °C (**e**,**f**).

Figure 5 shows the CL images taken at 540 nm corresponding to the previous SEM images and local CL spectra taken on locations indicated by the arrows in the images for untreated YAG:Ce^3+^ (a,b), C@YAG:Ce^3+^-1500 °C (c,d), and C@YAG:Ce^3+^-1650 °C (e,f). For untreated YAG:Ce^3+^, the 540 nm emission is relatively well distributed among the particles. The sharp bands at 320 nm are just occasionally observed, a fortiori mainly near the edge of the particles. For C@YAG:Ce^3+^-1500 °C, some areas show a very strong 540 nm emission, which is much higher than that of untreated YAG:Ce^3+^, while a very weak in other parts. It is noted that the relative emission intensity and spectral shape found in CL are slightly different compared to those found in PL, which may be related to the differences between light and e-beam irradiation and/or to optical detection differences. As already mentioned, UV and blue emissions are also observed, suggesting the existence of a Ce^3+^-doped secondary phase. The purple blue emission shows a maximum at 395 nm with a secondary maximum at 425 nm. Such doublet emission with an energy difference of about 1780 cm^−1^ is characteristic of the spin-orbit splitting of the 4f^1^ ground state of Ce^3+^ into a doublet (^2^F_7/2_ and ^2^F_5/2_). For C@YAG:Ce^3+^-1650 °C, the 540 nm emission is relatively uniformly distributed. In addition, two weak emission bands at 310 and 350 nm are observed, depending on the measured locations. It may be noted that contrary to untreated YAG:Ce^3+^, the UV emissions are more commonly observed.

**Figure 6:**
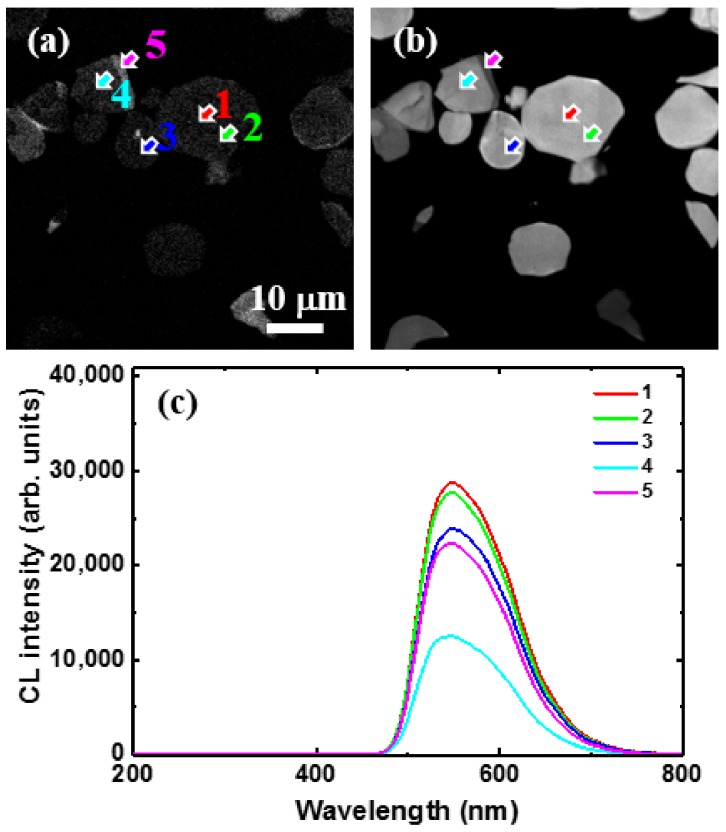
Cross-sectional CL images taken at 330 nm (**a**); and 540 nm (**b**) for untreated YAG:Ce^3+^; (**c**) Local CL spectra taken on locations indicated by arrows in the images.

In order to identify these additional bands, we have investigated the luminescence and chemical distribution by CL and EDS imaging on cross-sectioned samples. Figure 6 shows the cross-sectional CL images taken at 330 nm (a) and 540 nm (b) for untreated YAG:Ce^3+^. The 540 nm emission is well distributed in the particles, while UV emission slightly more at the edge of the particles. The EDS measurements did not reveal any particular elemental distribution. 

**Figure 7:**
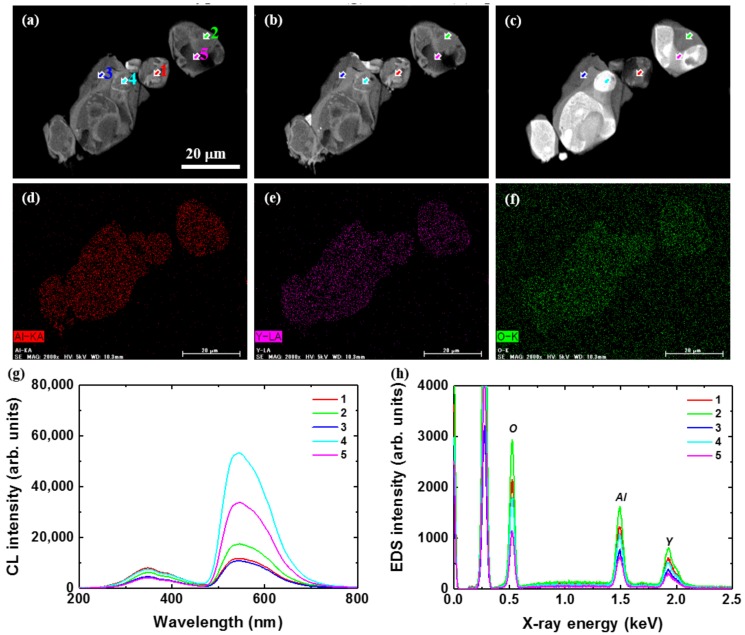
Cross-sectional CL images taken at 330 (**a**); 395 (**b**) and 540 nm (**c**) and Al (**d**); Y (**e**) and O (**f**) distributions for C@YAG:Ce^3+^-1500 °C/Type A; Local CL (**g**) and Energy-Dispersed Spectroscopy (EDS) (**h**) spectra taken on locations indicated by arrows in the images. In the EDS spectra, the first peak at 0.25 keV is ascribed to the carbon from epoxy resin in the pre-treatment of cutting before doing cross-sectional CL measurements.

During cross-sectional CL observations, it was found that the C@YAG:Ce^3+^-1500 °C areas could be categorized into two types: one presenting few large-size phases coexisting inside the particle, and another presenting many small areas with different luminescence properties. They will be called Type A and Type B, respectively, in the following. Figure 7 shows cross-sectional CL images taken at 330 (a), 395 (b), and 540 nm (c) and Al (d), Y (e) and O (f) distributions for C@YAG:Ce^3+^-1500 °C/Type A. The CL image taken at 540 nm indicates that the particles show the clear presence of large areas with different intensity. These patches seem to follow a core-shell distribution. However, it should be noted that the centre is the brightest for some particles and darkest for others. The CL images at 330 and 395 nm do not show such distribution, being comparatively well-distributed. No particular Al, Y, or O distributions are observed. Local CL (g) and EDS (h) spectra were taken on arrows indicated in the images. All of the CL spectra consist of the same bands: a broad band at 350 nm and the 540 nm band. Interestingly, while the 350 nm intensity is comparable for all of the positions, the intensity of the 540 nm band is 2–5 times higher for the spectra taken from the bright parts of the 540 nm image when compared to those from the dark ones. EDS spectra are similar to each other, all of which are consisting of Al, Y, and O. 

**Figure 8:**
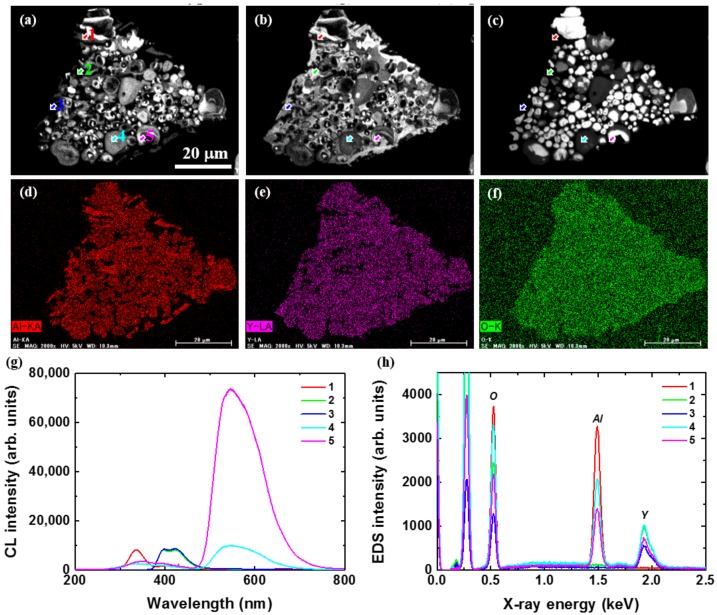
Cross-sectional CL images taken at 330 (**a**); 395 (**b**) and 540 nm (**c**) and Al (**d**); Y (**e**) and O (**f**) distributions for C@YAG:Ce^3+^-1500 °C/Type B; Local CL (**g**) and EDS (**h**) spectra taken on locations indicated by arrows in the images. In the EDS spectra, the first peak at 0.25keV is ascribed to the carbon from epoxy resin in the pre-treatment of cutting before doing cross-sectional CL measurements.

Figure 8 shows cross-sectional CL images taken at 330 (a), 395 (b) and 540 nm (c) and Al (d), Y (e), and O (f) distributions for C@YAG:Ce^3+^-1500 °C/Type B. The CL image taken at 540 nm shows the presence of many small patches inside the particle. Most of these patches have a diameter of ~2 μm. Particles also show the presence of different areas with different intensity. The 395 nm image seems complementary to that at 540 nm, while the 330 nm image seems to be more intermediate between the 395 and 540 nm images. It suggests that there is the coexistence of three phases inside the particles, with the one responsible for the 330 nm emission being at the interface between the two others. By EDS imaging, it is found that Al and Y elements are distributed in a complementary way. It seems that the Al distribution is more in agreement with the 330 nm image. To confirm these observations, local CL and EDS spectra have been taken on arrows indicated in the images, as shown in Figure 8g,h, respectively. Three types of CL spectra can be clearly identified. The CL spectrum from point 1 mainly consists of a broad band at 330 nm, while the EDS spectrum shows that it is Al-rich and Y-poor. The CL spectra from points 2 and 3 mainly consist of the doublet emission at 395 and 425 nm, while the EDS spectra show that these regions are Al-poor and Y-rich. B element is hardly detected due to its low sensitivity factor. Finally, the CL spectra from points 4 and 5 consist of the main YAG:Ce^3+^ emission with secondary bands at 350 and 395 nm, while the EDS spectra show that these regions are Al- and Y-rich. As for the origin of doublet emission at 395 and 425 nm, it should not come from the luminescence of AlN:Ce^3+^, which shows excitation peak at 280 nm and emission peak at 473 nm in the PL spectra [32]. Based on these observations and literature information, we can attribute the 330 nm emission to defects in Al_2_O_3_-like phase (F^+^ center) [33], 350 nm to defects in YAG:Ce^3+^ (Y_Al_ antisite defects, i.e., Y_Al_ AD) or to AlN (N vacancy or O interstitial, i.e., V_N_, O_i_) [34,35,36], and 395–425 nm doublet emission to Ce^3+^ in YBO_3_ phase [37]. It is interesting to note that the emission from the patches observed in the 540 nm image have a much stronger intensity when compared to those of the untreated YAG:Ce^3+^ or the C@YAG:Ce^3+^-1500 °C/Type A, suggesting a modification of the YAG:Ce^3+^phosphor, such as a reduction of residual Ce^4+^ into Ce^3+^ due to the strong reducing power of C. It is reasonably supposed that some reactions are induced at 1500 °C between C layer and YAG particles, and that the C@YAG:Ce^3+^-1500 °C/Type A and B correspond to different intermediate states of this reaction.

**Figure 9:**
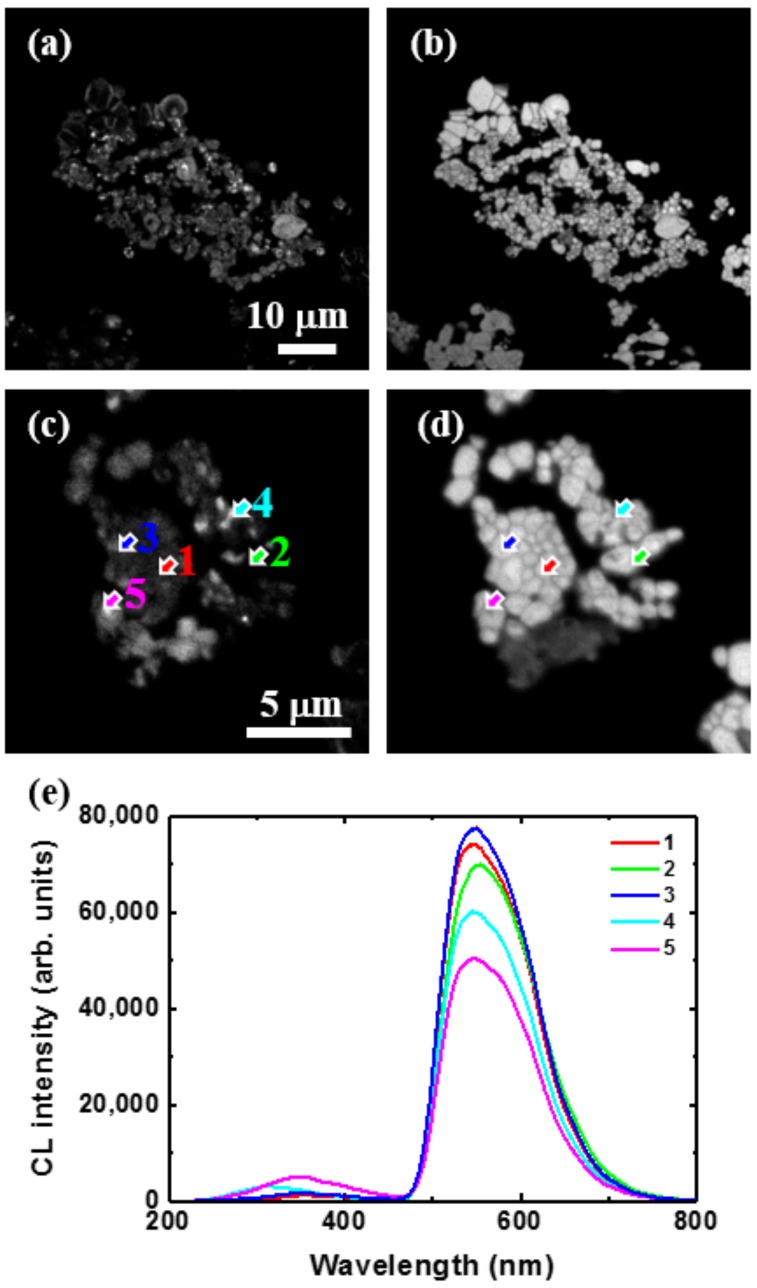
Cross-sectional CL images taken at 330 and 540 nm for C@YAG:Ce^3+^-1650 °C at x2000 (**a**,**b**) and ×7000 (**c**,**d**) magnifications, respectively; (**e**) and, Local CL spectra taken on locations indicated by arrows in the images.

Figure 9 shows the cross-sectional CL images taken at 330 (a,c) and 540 nm (b,d) for C@YAG:Ce^3+^-1650 °C at different magnifications. The 540 nm images reveal that the aggregated particles consist of uniformly bright small grains of about 1 um size separated by weakly luminescent grain boundaries. Namely, the C@YAG:Ce^3+^-1650 °C particles consist in reality of aggregates with few microns diameter. The 330 nm images show that the UV emission is more intense at the edge of the bigger particles, while it is more uniformly distributed for the smaller ones. Local CL spectra shown in Figure 9e show that the main bands are the Ce^3+^ emission in YAG:Ce^3+^ (at about 540 nm), and two small bands at 310 and 350 nm. The exact nature of the 310 nm emission is not clear, but we can presume that it is related to defects in YAG or the secondary phases. The intensity of Ce^3+^ emission is comparable to those found for C@YAG:Ce^3+^-1500 °C/Type B. The EDS measurements did not reveal any particular elemental distribution.

## 4. Discussion

Based on the above results, it was found there are different luminescent characteristics involved in the YAG:Ce^3+^, C@YAG:Ce^3+^-1500 °C, and C@YAG:Ce^3+^-1650 °C samples. Undoubtedly, the 540 nm emission in all of the samples is due to the well-known 5d-4f transition of Ce^3+^ in the YAG host lattice. It is still confusing about the origin of the UV emissions. Since Blasse and Bril reported the presence of a UV emission band besides the yellow emission of YAG:Ce^3+^ under cathode-ray excitation in 1967 for the first time [3], there were many papers published which discuss these UV emissions. According to these reports, the nature of the UV emission in YAG:Ce^3+^is related to radiation of an exciton localized around Y_Al_ AD (LE(AD) centers), by recombination luminescence of Y_Al_ AD or by the luminescence of F^+^ centers. To make an overview of the observed UV emissions in this paper, Table 1 summarizes all of the emission peaks and possible origins.

From PL and CL emission spectra, we found an increase of the intensity of YAG:Ce^3+^ for C@YAG:Ce^3+^-1650 °C, the appearance of a blue Ce^3+^-related emission at 395–425 nm for C@YAG:Ce^3+^-1500 °C, and changes in the luminescent centres responsible for UV emissions for the different samples. XRD, HRTEM, SEM-CL and SEM-EDS observations have revealed that the C@YAG:Ce^3+^-1500 °C particles consist of several phases coexisting inside the same particles, while C@YAG:Ce^3+^-1650 °C particles are composed of strongly aggregated luminescent submicron YAG:Ce^3+^ grains. Ce^3+^-doped YAG is considered to be stable at high temperatures, which is confirmed by XRD and PL analyses on uncoated YAG:Ce^3+^ heated at 1500 and 1650 °C in N_2_ atmosphere, as shown in Figure 1 and supporting information (Figures S1 and S2).

**Figure 10:**
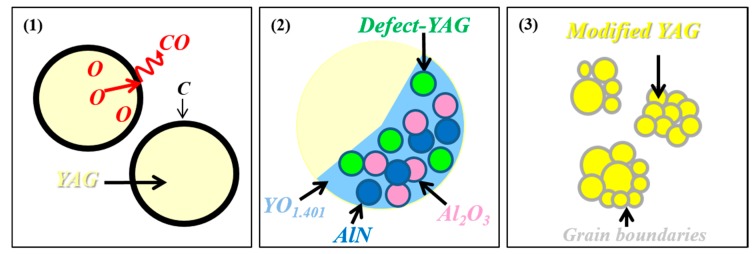
Schematic illustration of reaction process between C and YAG:Ce^3+^. (**1**) In the beginning, C reacts with YAG:Ce^3+^, which induces the formation of oxygen vacancies or induces carbothermal reduction and nitridation reactions; (**2**) Part of non-stoichiometric YAG starts to be decomposed into Y_2_O_3_, Al_2_O_3_ and nitrided AlN, resulting in a distribution of various luminescent particles with different particle size in C@YAG:Ce^3+^-1500 °C; (**3**) As increasing the heat-treatment temperature, the decomposition of YAG:Ce^3+^ is enhanced, followed by the dissolution of formed YBO_3_ and AlN/Al_2_O_3_, forming modified YAG:Ce^3+^sample with aggregated particles consisting of small grains of about 1 um size in C@YAG:Ce^3+^-1650 °C.

Thus, the evolutions of the luminescence properties as a function of heat-treatment temperature are explained by a reaction between C and YAG:Ce^3+^ induced by heating, as illustrated by Figure 10. We may suppose that C will react with O of YAG to form gas, like CO in N_2_ atmosphere_,_ thus inducing the formation of oxygen vacancies or inducing carbothermal reduction and nitridation reactions. It would be expected that this reaction will occur at the interface between YAG and the C layer. However, the CL images for C@YAG:Ce^3+^-1500 °C clearly show that the complete particle is affected by such reaction. It may be due to the self-diffusion of oxygen in YAG, which was attributed to the migration of oxygen vacancies [41], or to diffusion along grain boundaries between the crystallites, which is faster than bulk diffusion. Gradually, YAG will become poorer and poorer in O, which will result in the formation of non-stoichiometric YAG. This situation may correspond to C@YAG:Ce^3+^-1500 °C /Type A. Then, consequently, YAG may become unstable, and start to be decomposed into Y_2_O_3_ (later transformed to YBO_3_ as a result of B_2_O_3_ impurity), Al_2_O_3_ and nitrided AlN via carbothermal reduction and nitridation, resulting in a distribution of various luminescent particles, as observed for C@YAG:Ce^3+^-1500 °C /Type B. A mixing of the types A and B is observed for the C-coated YAG:Ce^3+^ particles heated at 1500 °C. We can imagine that the reactions are dependent on the particle size and/or C thickness, which is most certainly not uniform among the YAG particles. Moreover, since the apparent size of the particles increases after 1500 °C heat-treatment, we may suppose that some YAG particles are fused together. It may be noted that some AlN was observed by HRTEM, which suggests that part of decomposed Al_2_O_3_ will react with C to form AlN through carbothermal reduction and nitridation in N_2_ atmosphere. By increasing the heating temperature, the reaction may be enhanced, suggesting that the situation observed for C@YAG:Ce^3+^-1650 °C corresponds to the most advanced state of the reaction. This hypothesis is in agreement with the fact that C@YAG:Ce^3+^-1500 °C/Type B shows the existence of YAG:Ce^3+^ areas of few μm diameter with a very strong 540 nm emission, which is comparable to those observed for C@YAG:Ce^3+^-1650 °C. In C@YAG:Ce^3+^-1650 °C, the 350 nm emission, attributed to defects in YAG or in AlN, is more uniformly distributed inside the particles, while the 540 nm emission redshifts and becomes stronger. These results suggest that the composition of YAG:Ce^3+^ change, probably related to the incorporation of N, and/or further reduction of Ce^4+^ into Ce^3+^ in the presence of C. In addition, no Al_2_O_3_ or Ce^3+^-doped YBO_3_ related emissions are observed for C@YAG:Ce^3+^-1650 °C. It indicates that Ce^3+^-doped YBO_3_ and AlN/Al_2_O_3_ dissolve again to form modified YAG:Ce^3+^.

Still, many follow-up investigations can be done in order to optimize these reactions, which can be promising ways to enhance the emission intensity of YAG:Ce^3+^, and more generally, other oxide phosphors such as aluminates and silicates, and to control their particle size. Interestingly, in addition, the present method shows potential to tune the overall emission colour by varying the proportion ratios between blue-emitting YBO_3_:Ce^3+^ and yellow-emitting YAG:Ce^3+^, which makes it possible to achieve white light with such a phosphor when excited by a UV-LED chip.

## 5. Conclusions

In the present work, it has been demonstrated that the composition and luminescence properties of YAG:Ce^3+^ phosphor can be modified through the reaction between YAG:Ce^3+^ and carbon. The carbon coating on surface of YAG:Ce^3+^ powders can be achieved by CVD, followed by heating C@YAG:Ce^3+^ at 1500 and 1650 °C in N_2_ atmosphere, which promotes carbothermal reduction and nitridation reactions. It has been found that the emission intensity of YAG:Ce^3+^ increases for C@YAG:Ce^3+^-1650 °C, and interestingly, a new blue emission band in the range of 395–425 nm is observed for C@YAG:Ce^3+^-1500 °C. By using local analysis, these results can be explained by a reaction between C and YAG, resulting in a decomposition of YAG into Y-rich and Al-rich phases. The most advanced step of this reaction is the formation of aggregates of fine YAG:Ce^3+^ particles with an enhanced Ce^3+^ emission. Consequently, the emission colour of the YAG:Ce^3+^ phosphors can be varied from yellow, white, and then back to yellow under UV excitation due to phase, particle size, and composition modifications with increasing heat-treatment temperature on C@YAG:Ce^3+^. Such reactions are expected to occur also for other oxide phosphors, which supports a new strategy to tune the emission colour and improve the luminescence intensity of other oxide phosphors.

## Figures and Tables

**Table 1:** Overview of the emission peaks and their possible origins in YAG:Ce^3+^,C@YAG:Ce^3+^-1500 °C, and C@YAG:Ce^3+^-1650 °C samples.

Sample	Emission Peak/nm	Origins	Ref.
YAG:Ce^3+^	320 (sharp)	Gd^3+^ impurity	[31]
	540	YAG: Ce^3+^	
C@YAG:Ce^3+^-1500 °C	330	F^+^ center in Al_2_O_3_	[33], This work
	350	Y_Al_ ADin YAG/V_N_,O_i_in AlN	[34,35,36]
	395	F^+^center in YAG	[34,38,39]
	395–425 doublet	YBO_3_: Ce^3+^	[37], This work
	540	YAG: Ce^3+^	
C@YAG:Ce^3+^-1650 °C	310	LE(AD) in YAG	[35,40]
	350	Y_Al_ AD in YAG	[34,35]
	540	YAG:Ce^3+^	

## Supplementary Materials

The following are available online at www.mdpi.com/1996-1944/10/10/1180/s1, Figure S1: PLE (λ_em_ = 540 nm) and PL (λ_ex_ = 460 nm) spectra of untreated YAG:Ce^3+^, YAG:Ce^3+^-1500 °C and YAG:Ce^3+^-1650 °C, Figure S2: XRD patterns for untreated YAG:Ce^3+^, YAG:Ce^3+^-1500 °C and YAG:Ce^3+^-1650 °C.

## References

[1] Pan Y.X., Wu M.M., Su Q. (2004). Tailored photoluminescence of YAG: Ce phosphor through various methods. J. Phys. Chem. Solids.

[2] Lu J., Prabhu M., Song J., Li C., Xu J., Ueda K., Kaminskii A.A., Yagi H., Yanagitani T. (2000). Optical properties and highly efficient laser oscillation of Nd: YAG ceramics. Appl. Phys. B.

[3] Blasse G., Bril A. (1967). A new phosphor for flying-spot cathode-ray tubes for color television: Yellow-emitting Y_3_Al_5_O_12_–Ce^3+^. Appl. Phys. Lett..

[4] Potdevin A., Briois V., Caperaa N., Santilli C.V., Chadeyron G., Mahiou R. (2016). A thorough spectroscopic study of luminescent precursor solution of Y_3_Al_5_O_12_: Tb^3+^: Influence of acetylacetone. RSC Adv..

[5] Wang H.M., Huang Z.Y., Lu Z.W., Wang Q.Y., Jiang J.S. (2016). Determination of the elastic and plastic deformation behaviors of Yb: Y_3_Al_5_O_12_ transparent ceramic by nanoindentation. J. Alloys Compd..

[6] Thu L.D., Trung D.Q., Lam T.D., Anh T.X. (2016). Fabrication of far red emission phosphors Y_3_Al_5_O_12_: Eu (YAG: Eu) by co-precipitation method. J. Electron. Mater..

[7] Hora D.A., Andrade A.B., Ferreira N.S., Teixeira V.C., Rezende M.V.D. (2016). X-ray excited optical luminescence of Eu-doped YAG nanophosphors produced via glucose sol-gel route. Ceram. Int..

[8] Peng J., Zhu J.H., Li T. (2016). Numerical simulation and optimization of beam quality of 2.1 μm Cr, Tm, Ho: YAG laser with symmetric spherical resonator based on gradient-reflectivity mirror. Opt. Commun..

[9] Schiopu V., Macrin M., Cernica I., Iancu O., Manea A., Schiopu P. (2007). Synthesis of yttrium aluminium garnet doped with cerium for application in a new generation luminescent lighting devices. Advanced Topics in Optoelectronics, Microelectronics, and Nanotechnologies III.

[10] Munoz-Garcia A.B., Seijo L. (2011). Ce and la single- and double-substitutional defects in yttrium aluminum garnet: First-principles study. J. Phys. Chem. A.

[11] Munoz-Garcia A.B., Pascual J.L., Barandiaran Z., Seijo L. (2010). Structural effects and 4f-5d transition shifts induced by La codoping in Ce-doped yttrium aluminum garnet: First-principles study. Phys. Rev. B.

[12] Jung K.Y., Lee H.W. (2007). Enhanced luminescent properties of Y_3_Al_5_O_12_: Tb^3+^, Ce^3+^ phosphor prepared by spray pyrolysis. J. Lumin..

[13] Yang H., Kim Y.S. (2008). Energy transfer-based spectral properties of Tb-, Pr-, or Sm-codoped YAG : Ce nanocrystalline phosphors. J. Lumin..

[14] Jang H.S., Bin Im W., Lee D.C., Jeon D.Y., Kim S.S. (2007). Enhancement of red spectral emission intensity of Y_3_Al_5_O_12_: Ce^3+^ phosphor via pr co-doping and tb substitution for the application to white leds. J. Lumin..

[15] Park K., Kim T., Yu Y., Seo K., Kim J. (2016). Y/gd-free yellow Lu_3_Al_5_O_12_:Ce^3+^ phosphor for white leds. J. Lumin..

[16] Li J.S., Sun X.D., Li X.D., Liu S.H., Zhu Q. (2015). (Y, Lu)AG: Ce phosphors synthesized by stearate melting method and their fluorescence properties. J. Inorg. Mater..

[17] Zhu Q.Q., Hao L.Y., Xu X., Agathopoulos S., Zheng D.W., Fang C.H. (2016). A novel solid-state synthesis of long afterglow, Si-N co-doped, Y_3_Al_5_O_12_: Ce^3+^ phosphor. J. Lumin..

[18] Qiang Y.C., Yu Y.X., Chen G.L., Fang J.Y. (2016). Synthesis and luminescence properties of Ce^3+^-doped Y_3_Al_3.5_Ga_1.5_O_12_ green phosphor for white leds. J. Lumin..

[19] Pasinski D., Zych E., Sokolnicki J. (2016). The effect of N^3-^ substitution for O^2-^ on optical properties of YAG: Ce^3+^ phosphor. J. Alloys Compd..

[20] Oya T., Okada G., Yanagida T. (2016). Scintillation properties of Lu_3_Al_5_O_12_ co-doped with Nd and Ce. J. Ceram. Soc. Jpn..

[21] Tsai C.-C. (2014). Color rendering index thermal stability improvement of glass-based phosphor-converted white light-emitting diodes for solid-state lighting. Int. J. Photoenergy.

[22] Yin L.-J., Dong J., Wang Y., Zhang B., Zhou Z.-Y., Jian X., Wu M., Xu X., van Ommen J.R., Hintzen H.T. (2016). Enhanced optical performance of BaMgAl_10_O_17_: Eu^2+^ phosphor by a novel method of carbon coating. J. Phys. Chem. C.

[23] Yin L.-J., Xu X., Yu W., Yang J.-G., Yang L.-X., Yang X.-F., Hao L.-Y., Liu X.-J. (2010). Synthesis of Eu^2+^-doped AlN phosphors by carbothermal reduction. J. Am. Ceram. Soc..

[24] Piao X.Q., Horikawa T., Hanzawa H., Machida K. (2006). Characterization and luminescence properties of Sr_2_Si_5_N_8_: Eu^2+^ phosphor for white light-emitting-diode illumination. Appl. Phys. Lett..

[25] Kuang J.C., Zhang C.R., Zhou X.G., Liu Q.C., Ye C. (2005). Formation and characterization of cubic aln crystalline in a carbothermal reduction reaction. Mater. Lett..

[26] Ozawa M., Loong C.K. (1999). In situ x-ray and neutron powder diffraction studies of redox behavior in CeO_2_-containing oxide catalysts. Catal. Today.

[27] Cho Y., Dierre B., Sekiguchi T., Suehiro T., Takahashi K., Takeda T., Xie R.-J., Yamamoto Y., Hirosaki N. (2016). Low-energy cathodoluminescence for (oxy)nitride phosphors. J. Vis. Exp..

[28] Lee C., Wei X., Kysar J.W., Hone J. (2008). Measurement of the elastic properties and intrinsic strength of monolayer graphene. Science.

[29] Cho Y.W., Charles J.A. (1991). Synthesis of nitrogen ceramic powders by carbothermal reduction and nitridation Part 3. Aluminum nitride. Mater. Sci. Technol..

[30] Shyu J.-J., Yang C.-W. (2017). Improvement of photoluminescence intensity of Ce-doped Y_3_Al_5_O_12_ phosphor by Si_3_N_4_ addition. Mater. Chem. Phys..

[31] Zorenko Y., Gorbenko V., Konstankevych I., Voloshinovskii A., Stryganyuk G., Mikhailin V., Kolobanov V., Spassky D. (2005). Single-crystalline films of Ce-doped YAG and LuAG phosphors: Advantages over bulk crystals analogues. J. Lumin..

[32] Hu W.-W., Zhu Q.-Q., Hao L.-Y., Xu X., Agathopoulos S. (2014). Luminescence properties and energy transfer in AlN: Ce^3+^, Tb^3+^ phosphors. Mater. Res. Bull..

[33] Colyott L.E., Akselrod M.S., McKeever S.W.S. (1996). Phototransferred thermoluminescence in alpha-Al_2_O_3_: C. Radiat. Prot. Dosim..

[34] Zorenko Y., Mares J.A., Prusa P., Nikl M., Gorbenko V., Savchyn V., Kucerkova R., Nejezchleb K. (2010). Luminescence and scintillation characteristics of YAG: Ce single crystalline films and single crystals. Radiat. Meas..

[35] Zorenko Y., Gorbenko V., Savchyn V., Vozniak T., Puzikov V., Danko A., Nizhankovski S. (2010). Time-resolved luminescent spectroscopy of YAG: Ce single crystal and single crystalline films. Radiat. Meas..

[36] Jadwisienczak W.M., Lozykowski H.J., Berishev I., Bensaoula A., Brown I.G. (2001). Visible emission from AlN doped with Eu and Tb ions. J. App. Phys..

[37] Nohara A., Takeshita S., Isobe T. (2014). Mixed-solvent strategy for solvothermal synthesisof well-dispersed YBO_3_: Ce^3+^,Tb^3+^ nanocrystals. RSC Adv..

[38] Zorenko Y., Zych E., Voloshinovskii A. (2009). Intrinsic and Ce^3+^-related luminescence of YAG and YAG: Ce single crystals, single crystalline films and nanopowders. Opt. Mater..

[39] Zorenko Y., Voznyak T., Gorbenko V., Zych E., Nizankovski S., Dan'ko A., Puzikov V. (2011). Luminescence properties of Y_3_Al_5_O_12_: Ce nanoceramics. J. Lumin..

[40] Zorenko Y., Voloshinovskii A., Savchyn V., Voznyak T., Nikl M., Nejezchleb K., Mikhailin V., Kolobanov V., Spassky D. (2007). Exciton and Antisite Defect-Related Luminescence in Lu_3_Al_5_O_12_ and Y_3_Al_5_O_12_ Garnets.

[41] Haneda H., Miyazawa Y., Shirasaki S. (1984). Oxygen diffusion in single crystal yttrium aluminum garnet. J. Cryst. Growth.

