# Supplementary material for: Transition of Emission Colours as a Consequence of Heat-Treatment of Carbon Coated Ce3+-Doped YAG Phosphors
Source: Materials (Basel). 2017 Oct 16;10(10):1180. doi: 10.3390/ma10101180 (PMC5666986; doi:10.3390/ma10101180)
Supplement: Supplementary file 1 [file materials-10-01180-s001.pdf]

Supplementary Materials

Transition of emission colours as a consequence of heat-treatment of carbon coated Ce^{3+}-doped YAG phosphors

Liang-Jun Yin [1,2,*,†], Benjamin Dierre [3,*,†], Takashi Sekiguchi [4], J. Ruud van Ommen [2], Hubertus T. (Bert) Hintzen [3], Yujin Cho [4,*,†]

1 School of Energy Science and Engineering, University of Electronic Science and Technology of China, 2006 Xiyuan Road, Chengdu 610051, China

2 Department of Chemical Engineering, Faculty of Applied Sciences, Delft University of Technology, Van der Maasweg 9, 2629 HZ Delft, The Netherlands; J.R.vanOmmen@tudelft.nl

3 Luminescent Materials Research Group, Faculty of Applied Sciences, Delft University of Technology, Mekelweg 15, 2629 JB Delft, The Netherlands; h.t.hintzen@tudelft.nl

4 Semiconductor Device Materials Group, International Center for Materials Nanoarchitectonics (MANA), National Institute for Materials Science (NIMS), 1-1 Namiki, Tsukuba, Ibaraki 305-0044, Japan; sekiguchi.takashi@nims.go.jp

* Correspondence: ylj@mail.ustc.edu.cn (L.J.Y.); b.f.p.r.dierre@tudelft.nl (B.D.); cho.yujin@nims.go.jp (Y.C.)

† These authors contributed equally to this work.

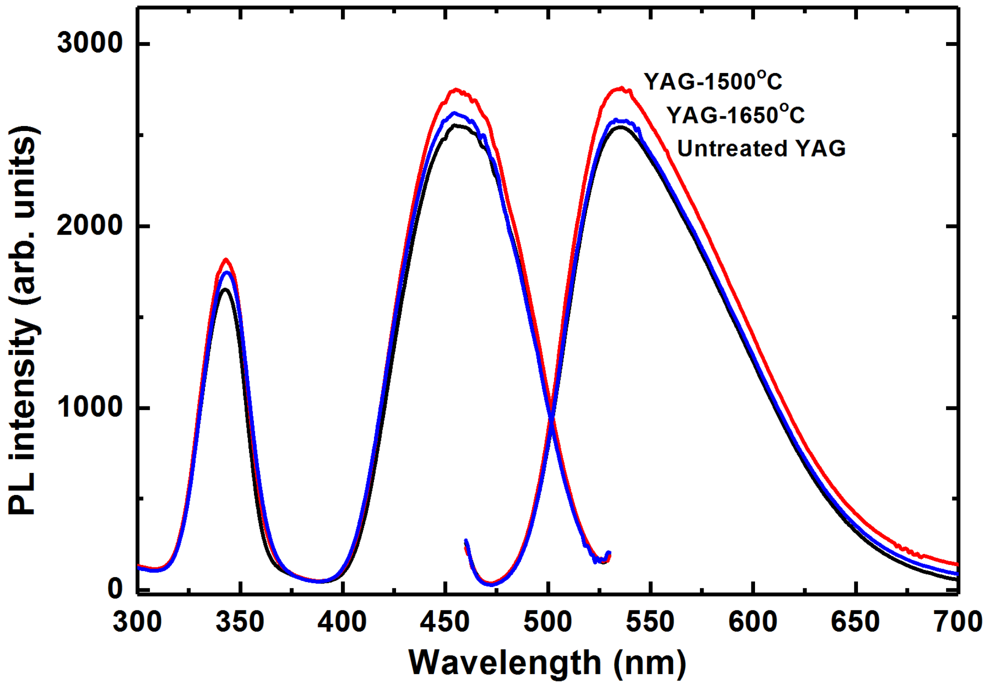

Figure S1. PLE (λ_{em}=540nm) and PL (λ_{ex}=460nm) spectra of untreated YAG:Ce^{3+}, YAG:Ce^{3+}-1500 ºC and YAG:Ce^{3+}-1650 ºC.

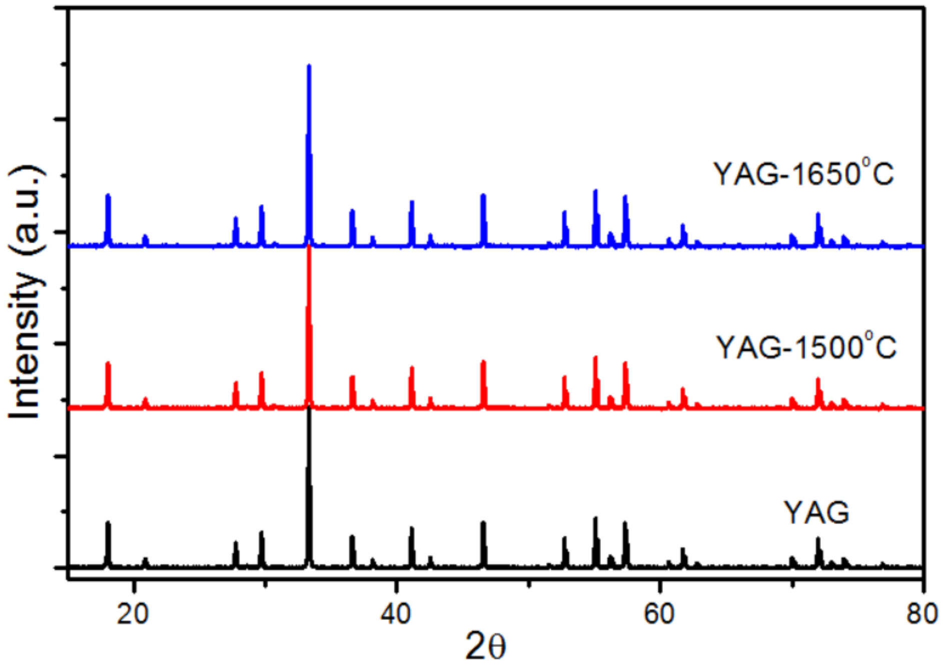

Fig. S2. XRD patterns for untreated YAG:Ce^{3+}, YAG:Ce^{3+}-1500 ºC and YAG:Ce^{3+}-1650 ºC.
